# Isolation and molecular confirmation of *Brucella suis* biovar 2 from slaughtered pigs: an unanticipated biovar from domestic pigs in Egypt

**DOI:** 10.1186/s12917-022-03332-2

**Published:** 2022-06-13

**Authors:** Walid Elmonir, Nour H. Abdel-Hamid, Mahmoud E. R. Hamdy, Eman I. M. Beleta, Mohamed El-Diasty, Falk Melzer, Gamal Wareth, Heinrich Neubauer

**Affiliations:** 1 Department of Hygiene and Preventive Medicine (Zoonoses), Faculty of Veterinary Medicine, Kafrelsheikh University, Kafrelsheikh, Egypt; 2 Agricultural Research Center, Animal Health Research Institute, P.O. Box 264-Giza, Cairo, 12618 Egypt; 3 Institute of Bacterial Infections and Zoonosis, Friedrich-Loeffler Institut, Naumburger Str. 96a, 07743 Jena, Germany; 4 Department of Bacteriology, Immunology, and Mycology, Faculty of Veterinary Medicine, Benha University, PO Box 13736, Toukh, Moshtohor Egypt

**Keywords:** *Brucella suis*, Prevalence, Suis ladder PCR, Atypical phenotype, Domestic pigs

## Abstract

**Background:**

*Brucella suis* is a zoonotic pathogen with a serious impact on public health and the pig industry worldwide. Information regarding *B. suis* in pigs in Egypt is scarce. This study aimed to investigate the prevalence of *B. suis* in slaughtered domestic pigs at El-Basatin abattoir in Cairo, Egypt. A total of 1,116 domestic pigs slaughtered in 2020 were sampled for *Brucella* isolation and identification. Identified *Brucella* isolates were molecularly confirmed at species, and biovar levels using Bruce ladder PCR and Suis ladder multiplex PCR. Additionally, high-risk practices of 16 abattoir workers (4 veterinarians, 10 butchering and evisceration workers, and 2 scalding workers) were investigated using a pre-piloted structured questionnaire.

**Results:**

*Brucella* isolates were recovered from 1.3% of examined pigs (*n* = 14) at consistently low rates (1.1—2.9%) across the year of sampling from February to December 2020. All isolates were confirmed as *B. suis* biovar (bv) 2. Remarkably, 92.9% (13/14) of isolates showed atypical ability to produce H_2_S and hence were considered as *B. suis* bv2 atypical phenotype. The prevalence was higher in males (1.8%) than in females (0.9). However, this difference was not significant (Odds ratio = 1.9; CI 95% 0.7 – 5.7; *P* = 0.2). No detectable pathological lesions were associated with *B. suis* bv2 infection in examined pigs. All strains were isolated from cervical lymph nodes, highlighting a potential oral transmission. High-risk practices were recorded among swine abattoir workers in this study: 75% do not wear gloves or disinfect their knives daily, and 18.8% were willing to work with open wound injuries.

**Conclusions:**

To the best of our knowledge, this is the first isolation of *B. suis* bv2 in Egypt. Detection of H_2_S producing *B. suis* bv2 atypical phenotype is alarming as it may result in misinterpretation of these isolates as highly human pathogenic *B. suis* bv1 in Egypt and possibly elsewhere. Further epidemiological tracing studies are crucial for the detection of the origin of this biovar. Including pigs in the national surveillance program of brucellosis, and an education program for swine abattoir workers about occupational risk of *B. suis* is a need in Egypt.

**Supplementary Information:**

The online version contains supplementary material available at 10.1186/s12917-022-03332-2.

## Background

*Brucella suis* (*B. suis*) is the causative agent of porcine brucellosis. Based on preferential infection of different animal hosts, it encompasses five biovars (bv1-5). Biovars 1, 2, and 3 infect domestic pigs, wild boars, and hares, bv 4 is restricted to reindeer and caribou, and bv 5 infects rodents only [1]. Biovar 1 is widely spread in Central and South American pigs, causing several brucellosis outbreaks in pigs, and is linked to zoonotic human infections [2, 3]. Biovars 1 and 3 cause sporadic epidemics in Asia, especially in China [3]. Biovar 4 is endemic to Alaska, Canada, and Northern Russia. It was reported as a rare cause of human disease [4]. On the contrary, biovar 2 is restricted to Europe [5], where both wild boar (*Sus scrofa scrofa*) and European brown hare (*Lepus europaeus*) are the only recognized wildlife reservoirs of this biovar [3, 6]. Recently, an emerging increase of biovar 2 brucellosis outbreaks in pigs was reported in Europe. These outbreaks mainly were linked to contact of domestic pigs with wildlife reservoirs [3, 5, 6], import of carrier pigs, or use of infected boar semen [5]. Additionally, spillover infections spread to cattle were reported in several European countries [7, 8]. *B. suis* bv 2 causes bacteremia, abortion, stillbirths, decreased litter size, weak piglets, infertility, and sometimes chronic focal localization as abscess formation in various organs, metritis in females, orchitis in males, and swollen joints with lameness [3, 9]. *B. suis* bv 2 outbreaks have been associated with a high economic impact on the pig industry in Europe [5].

Nine human brucellosis cases caused by *B. suis* bv2 were reported globally; eight were in France [10, 11], and one case was in China [12]. Six out of these eight human cases reported in France had chronic medical conditions that possibly increased their risk of infection [11]. However, under-reporting is expected since most European countries, where biovar 2 is enzootic in wild pigs, do not routinely full type *Brucella* in human cases [11]. Three of these cases presented with acute brucellosis without focal infection, three cases suffered from arthritis, and two cases were associated with abscesses formation in muscles and liver [11, 12]. One patient died from liver failure [12]. The majority of these cases were linked to frequent exposure to wild boars or their tissues (e.g., hunting, butchering, etc.), which highlights the occupational hazard of this biovar [11].

In Egypt, *B. suis* is an uncommon cause of brucellosis, while *B. melitensis* bv3 and *B. abortus* bv1 have been reported in the majority of human and animal brucellosis cases [13–15]. Since the first isolation of *B. suis* in 1942 from aborted sows in upper Egypt [16], only a few recent reports of *B. suis* in pigs have been published [17, 18]. Pigs are not included in the routine national brucellosis surveillance program, which contributes to the lack of knowledge about the epidemiology of *B. suis,* its economic impact, or the role of pigs in spreading the infection to other livestock or humans. Pigs were reared in Egypt on intensive large-scale pig farms and small-scale backyard husbandry by garbage collectors in the slums of Cairo and Giza governorates, who raised pigs as the main source of their income [19]. However, in 2009 the General Organization of Veterinary Services (GOVS) ordered the culling of pig populations for fear of transmission of swine influenza virus to humans [19]. Pig slaughtering was permitted again in 2014. Despite the pig rearing ban from 2009 to 2014, garbage collectors managed to smuggle some of their pigs into their homes and reared them hidden from the GOVS. After 2014, the entire pig production stayed with these small-scale breeders, as former large-scale pig farmers refused to reopen their farms in fear of other severe economic losses. In small-scale backyard farms, pigs are fed on organic garbage escaping veterinary supervision and lack biosecurity measures but have contact with other livestock [18, 20]. Notably, on some occasions, pigs of these backyard farms are sold to small breeders in other northern (e.g., Alexandria) and southern (e.g., El-Minia) governorates, which highlights the risk of spreading porcine brucellosis to other livestock and across the country. In support of this assumption, *B. suis* was recovered from cattle in northern regions [20] and from camels in the southern region of Egypt [21]. Thus, this study was done to investigate the prevalence of *B. suis* in slaughtered pigs by isolation and molecular identification of its circulating biovars in El-Basatin abattoir and to record the high-risk practices of abattoir workers using a pre-tested questionnaire.

## Results

A total of 14 *Brucella* isolates were recovered from 1,116 slaughtered pigs (1.3%). Using Suis-ladder multiplex PCR, all 14 *Brucella* isolates were identified as *B. suis* bv2. The conventional bacteriological examinations showed that 13 isolates were smooth, and one isolate was a rough strain (ID 14, Table 1). Also, all isolates showed the same characteristics, including agglutination with anti-*Brucella* monospecific anti-A serum (except for the rough strain; ID 14), positive reactions in urease test (fast; within a few minutes), growth without CO_2_, no growth on thionin (1/25,000) or basic fuchsin dye (20 µg/ml), and lysis by TB phage only at a concentration of 10^4^ × RTD (Table 1). These characteristics are typical for *B. suis* biovar 2. However, the 13 smooth isolates showed an atypical ability to produce H_2_S that was only reported in *B. suis* bv1 (Table 1). Therefore, these isolates were assigned as *B. suis* bv2a (Table 2). The *B. suis* bv2 isolates were recovered at consistently low rates (1.1—2.9%) across the year of sampling from February to December 2020 (Table 2). The prevalence and odds of *Brucella* isolation from males were higher than from females (1.8% vs. 0.9%, respectively; OR = 1.9; CI 95% 0.7 – 5.7). However, these differences were insignificant (*P* = 0.2) (Table 3). This study had no detectable pathological lesions in slaughtered pigs infected with *B. suis* bv2. Among the examined lymph nodes samples, *B. suis* bv2 isolates were only recovered from the cervical lymph node (LN).

**Table 1:** Diagnostic characteristics of *Brucella suis* isolates from slaughtered pigs in this study

ID	Genus specific PCR	MALDI-TOF Analysis	AMOS-PCR	Suis-ladder multiplex PCR	H_2_S production	Agglutination with Monospecific antisera	
						**A**	**M**
1	+	*Brucella* spp	-	*B. suis* bv 2	+	+	-
2	+	*Brucella* spp	-	*B. suis* bv 2	+	+	-
3	+	*Brucella* spp	-	*B. suis* bv 2	+	+	-
4	+	*Brucella* spp	-	*B. suis* bv 2	+	+	-
5	+	*Brucella* spp	-	*B. suis* bv 2	+	+	-
6	+	*Brucella* spp	-	*B. suis* bv 2	+	+	-
7	+	*Brucella* spp	-	*B. suis* bv 2	+	+	-
8	+	*Brucella* spp	-	*B. suis* bv 2	+	+	-
9	+	*Brucella* spp	-	*B. suis* bv 2	+	+	-
10	+	*Brucella* spp	-	*B. suis* bv 2	+	+	-
11	+	*Brucella* spp	-	*B. suis* bv 2	+	+	-
12	+	*Brucella* spp	-	*B. suis* bv 2	+	+	-
13	+	*Brucella* spp	-	*B. suis* bv 2	+	+	-
14^R^	+	*Brucella* spp	-	*B. suis* bv 2	-	-	-

All *B. suis* isolates were positive for the Urease test (rapid 1 min) and negative for growth on Thionine agar (1/25,000); ^R^: Rough strain; + : Positive; -: Negative

**Table 2:** Temporal frequencies of detected *B. suis* bv2 isolates from slaughtered pigs in this study

Isolate ID	Confirmed species/biovar	Sampling groups date	Samples No. / group	Positive no./ group	Prevalence / Month
1	*B. suis* bv2a	19/02/2020	90	1	**1.1 (1/90)**
2	*B. suis* bv2a	16/07/2020	100	1	**2 (5/250)**
3	*B. suis* bv2a	26/07/2020	150	4	
4	*B. suis* bv2a				
5	*B. suis* bv2a				
13	*B. suis* bv2a				
-	-	16/08/2020	90	-	-
-	-	05/09/2020	100	-	-
6	*B. suis* bv2a	18/10/2020	156	6	**2.9 (8/276)**
9	*B. suis* bv2a				
7	*B. suis* bv2a				
8	*B. suis* bv2a				
10	*B. suis* bv2a				
11	*B. suis* bv2a				
12	*B. suis* bv2a	22/10/2020	120	2	
14	*B. suis* bv2				
-	-	19/11/2020	110	-	-
-	-	01/12/2020	102	-	-
-	-	10/12/2020	98	-	-

*B. suis* bv2a: Strains with atypical H_2_S production phenotype; -: Negative

**Table 3:** Frequency/distribution of *Brucella suis* bv2 in both sex of slaughtered pigs in Cairo, Egypt

Pathogen	Category	Prevalence			OR	95% CI	*P*_-value_
		**No**	**Pos**	**%**			
***B. suis* bv 2**	Female	663	6	0.9	-	-	-
	Male	453	8	1.8	1.9	0.7 – 5.7	0.2
**Total**		1116	14	1.3			

This study recorded high-risk practices at high rates among swine abattoir workers (Table 4). For example, none of the workers would wear masks, goggles, or face shields while working, and only 25% would wear gloves. In addition, some workers would work with open hand wounds (18.8%) and smoke while working (37.5%). Finally, only four workers (4 veterinarians, 25%) would disinfect their knives daily.

**Table 4:** High-risk practices of workers in Basateen pig abattoir in Egypt

Topics		Category	Yes (%)	No (%)
**Identification**	**Gender**	Male	15 (93.8)	0 (0)
		Female	1 (6.3)	0 (0)
	**Education**	illiterate	12 (75)	0 (0)
		Bachelor	4 (25)	0 (0)
	**Residence**	Urban	11 (68.8)	0 (0)
		Rural	5 (31.3)	0 (0)
	**Occupation**	Vets	4 (25)	0 (0)
		^a^Other workers	12 (75)	0 (0)
**Practices**	**Using PPE**		16 (100)	0 (0)
	**If using PPE, kinds used**	Gloves	4 (25)	12 (75)
		Mask	0 (0)	16 (100)
		Goggles	0 (0)	16 (100)
		Face shield	0 (0)	16 (100)
		Aprons	6 (37.5)	10 (62.5)
		Boots	16 (100)	0 (0)
	**Get injured during working**		16 (100)	0 (0)
	**Working with open/cut hand-wound**		3 (18.8)	13 (81.3)
	**Eating while working**		0 (0)	16 (100)
	**Smoking while working**		6 (37.5)	10 (62.5)
	^b^**Washing hands before eating or smoking**		6 (100)	0 (0)
	^b^**If yes, using soap in hand wash**		0 (0)	6 (100)
	**Disinfecting knives daily**		4 (25)	12 (75)

^a^Other workers include those working in butchering, evisceration, and scalding (dehairing)

^b^these questions were answered by respondents who eat or smoke while working

## Discussion

In Egypt, pigs are raised and consumed mainly by Christians and tourists due to religious constrictions on Muslims, the majority of the population. To the best of our knowledge, this is the first report of *B. suis* bv2 from pigs in Egypt. Despite very early detection of *B. suis* in sows with reproduction failure in upper Egypt in 1942 [16], records of *B. suis* detection in livestock in Egypt are very limited. *B. suis* isolates were previously detected in seven slaughtered pigs from Cairo and Giza governorates [17] and in two cows from Menofia and Beni-Suef governorates [20]. In these two studies, *B. suis* isolates were typed as biovar 1 (*B. suis* bv1) either phenotypically [17] or genetically [20]. In another recent study, the DNA of *B. suis* was detected in the blood of three slaughtered pigs; however, the biovar type was not determined [18]. Remarkably, *B. suis* bv2 was never reported in Egypt or other counties outside Europe. *B. suis* bv2 is exclusively reported in Europe, where spillover infections from wild reservoirs have resulted in emerging outbreaks in domestic pig herds in several European countries [3, 5].

Pigs were reared, and pork was consumed all over Egypt in ancient time. Thus, it can be assumed that porcine brucellosis had been present in the past and reintroduced with imported pigs in the recent past or with the import of subclinically infected animals. Therefore, the transmission of Egyptian hares might have resulted as well.

Egypt was self-sufficient in the pig industry as national pig production covers national market needs in the years leading up to 2009. After the 2009 governmental mass culling of more than 300,000 pigs due to the fear of the spread of the swine flu virus [19], the national market needs have been covered by the importation of frozen pork and other pork byproducts but not living pigs. In fact, during the 2009 crisis, the GOVS firmly prohibited the importation of live pigs, banned breeding or keeping pigs across the country, and secured the borders to prevent the potential smuggling of pigs into or out of Egypt. Additionally, there was no official report of live pigs’ importation in the last 20 years. For these reasons, the possible introduction of the *B. suis* bv2 in Egypt through official imported infected pigs can be ruled out.

Before boar semen importation was officially prohibited in 2009, only a few large-scale pig breeders reported using unofficially imported boar semen to improve the genetic production traits of their pig stocks (personal communication; Airport vet quarantine, Veterinarians working at the pig slaughterhouse, and pig breeders). We could not identify the source of the imported semen as these breeders imported the semen through private companies which are very difficult to trace. Therefore, there is no clue how or when *B. suis* bv2 strains entered Egypt using official routes.

Pig farming is the main source of income for the garbage collectors living in the slums of Cairo and Giza governorates; hence pig culling left thousands of garbage collectors jobless. Also, the majority of local customers (low-income families) did not buy expensive (and less tasty) imported frozen pork as they relied on cheap local pig meat as the main source of protein [19]. These demanding local needs encouraged many garbage collectors, who succeeded in hiding some of their pigs from culling, to rebuild their small-scale pig farming business. In April 2014, the GOVS permitted the reopening of the pig slaughter section in El-Basatin abattoir, a milestone for pig farmers and the local pig industry. Since then, small-scale pig farmers have been thrilled to increase their pigs’ stocks to cover the rising market needs. This rapid increase in pig numbers (6000 heads in 2014 to 25,000 heads in 2018) over a short period [22] raised concerns about the restocking of the population via illegal routes to improve litter numbers, litter sizes, and meat production performance by the introduction of superior European breeds. Several African countries have imported pigs or boar semen to improve local breeds [23–25]. Another scenario is that the potential informal import of pigs or semen from other African countries lacking proper veterinary public health structures is an imminent risk for the pig industry in Egypt.

Only 11 of 54 African countries conducted targeted surveillance for *Brucella* in pigs (2007–2020) [26]. Accordingly, *B. suis* is believed to be widespread yet underreported in Africa [3, 27]. Moreover, *B. suis* spillover infections have been reported from atypical hosts such as cattle [20, 28] and rodents [27], indicating well-established *B. suis* reservoirs in Africa. *B. suis* bv2 may have entered Africa by pig/semen import and become established, but unidentified, among domestic pig breed or wildlife reservoirs a long time ago. Cross-border live animal or semen trade, either formal or informal, is very common in Africa, and control of animal movement across borders is very limited [23]. Egypt shares borders and makes trades with Sudan, a country with cross-border trade with Uganda, Rwanda, and Kenya. These countries imported boar semen from Europe in the past [23]. As the transmission of infections across the border from Sudan to Egypt is not unfamiliar, and recently *B. suis* DNA detection (unknown biovar) in camels’ sera in a southern governorate of Egypt was attributed to possible transboundary transmission of *B. suis* infection from Sudanese pig farms [21]. The assumed import of brucellosis via this trade route is also supported by an informal report (possibly through the private sector) stating that Egypt imported 32 pigs from Sudan in 2016 [29].

Whatever the potential routes of entry of *B. suis* bv2 infection to Egypt are, our results suggest that this introduction is not recent. We could detect *B. suis* bv2 all over the year of our sampling and at a relatively persistent low prevalence (1.1%—2.1% from February to October 2020). This highlights that *B. suis* bv2 infection is established in several pig herds in Egypt. Further genetic studies e.g., whole-genome sequence analysis, may add information on the origin of these isolates and help to trace their sources.

Remarkably, 13 of the detected *B. suis* bv2 isolates showed atypical ability to produce H_2_S that was only reported in *B. suis* bv1 [30]. This is a critical finding since H_2_S production is used to differentiate *B. suis* bv1 from other *B. suis* biovars (negative on H_2_S production); hence, mis- or under-reporting of these variants of *B. suis* bv2 is expected in Egypt and possibly elsewhere. These findings recommend routine use of molecular confirmation for both species and biovar in routine identification of *Brucella* isolates. *B. suis* isolates with atypical phenotype were previously reported, mostly for dye resistance [2, 31]. This finding, however, points to a single source of infection, e.g., active trade of semen or pigs. Whole-genome sequencing will facilitate a better understanding of this finding as well.

None of the positive pigs for *B. suis* bv2 showed visible pathological lesions in this study. This could be attributed to the young age of slaughtered animals, as most of the pigs were five months old; young animals rarely demonstrate clinical signs of *B. suis* infection [3, 32]. Some studies suggested that the long incubation period of the disease and/or the lack of sexual maturity were the reasons for the lack of clinical signs in younger pigs [3, 32]. In some instances, even mature pigs may show no clinical signs when infected with *B. suis* [3, 33]. There was no significant difference in prevalence between males and females (*P* = 0.2). This finding also can be explained by the age of the slaughtered pigs: most animals were not sexually mature. Differences are more evident in mature animals, as *B. suis* tends to localize more frequently and for a longer time in reproductive tissues of boars than in female ones [3]. All *B. suis* bv2 isolates were recovered from cervical LN. The cervical LNs are selective niches for localization of *Brucella* spp. in case of oral infection; *Brucella* strains may persist for up to two months in these lymph nodes after infection [34]. This highlights that oral exposure via feed (or environment) contaminated by reproductive discharges of infected sows was the predominant intra-herd transmission route among this study's slaughter pigs. This is expected since these young immature animals were reared for fattening, not breeding. Also, the majority of these animals did not reach sexual maturity, so venereal transmission and localization in genital lymph nodes are deemed unlikely.

The *B. suis* infection is an occupational risk for abattoir workers [35]. Human clinical cases caused by *B. suis* bv2 were reported from Europe [10, 11] and elsewhere [12]. The majority of these cases had a high risk of infection as they were often exposed to infection because of their occupation (e.g., skinning and gutting hunted wild boar) [11]. Furthermore, most abattoir workers in this study did not wear masks or gloves or disinfect their knives (75—100%). This highlights the potential high risk of infection among abattoir workers in the study region for brucellosis caused by *B. suis* or other *Brucella* spp. found in pigs.

## Conclusions

This is the first report of isolation and molecular confirmation of *B. suis* bv2 in domesticated pigs in Egypt. Scenarios about possible introduction via informal or illegal smuggling of living pigs or via boar semen need further studies, including whole-genome sequence (WGS) analysis. The detection of *B. suis* bv 2 at consistently low rates across the year suggests a long-established reservoir rather than a recent introduction of foreign strains. Detection of H_2_S producing *B. suis* bv2 atypical phenotype is alarming as it may result in misinterpretation of these isolates as highly human pathogenic *B. suis* bv1 in Egypt and possibly elsewhere. Thus, the use of the molecular approach for *B. suis* biovar identification should be mandatory to detect these atypical phenotype isolates if stable genetic markers can be identified. Finally, the study showed that pigs reared in slums could be a neglected source for spillover infections to humans or other animals with *B. suis* and possibly other *Brucella* species in Egypt. This finding necessitates immediate and regular surveillance of these small-scale pig herds by GOVS authorities for early detection and control of *B. suis* biovars spread in Egypt.

## Materials and methods

### Study area

**Fig. 1:**
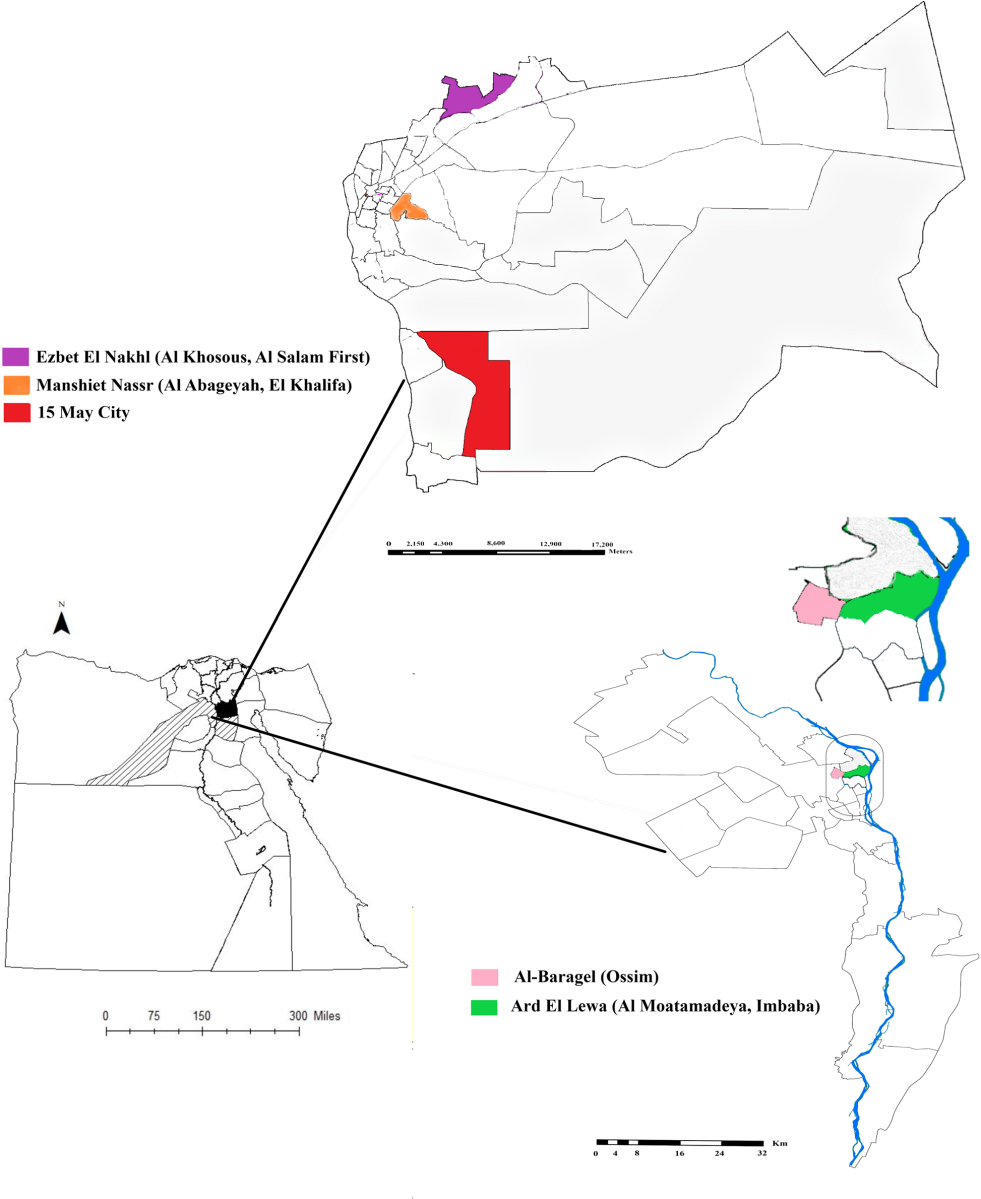
Locations of backyard reared pig herds in slums of Cairo and Giza governorates, Egypt

Pig samples were collected from slaughtered pigs in El-Basatin abattoir in the southern region of Cairo governorate. The GOVS permitted pig slaughtering in El-Basatin abattoir in April 2014 after the official banning of pig rearing and slaughtering in 2009. All pigs sent for slaughtering in El-Basatin abattoir were raised in the slums of Cairo and Giza governorates by garbage collectors as one of their main sources of income. In these slums, pigs are reared in backyard pens containing 50 to 100 pigs. Five large slums in Cairo and Giza governorates are currently raising pigs. The Cairo governorate slums include Manshiet Nassr, Cairo's largest pig farming slum, in the western Cairo region (30.0362°N, 31.2783°E); Ezbet El Nakhl (30.1393°N, 31.3244°E) in eastern Cairo region; and 15 May city (29.8579°N, 31.3885°E) in southern Cairo region. The Giza governorate slums included Al-Baragel (30.0767°N, 31.1593°E); and Ard El Lewa (30.0544°N, 31.1854°E) (Fig. 1). Most pigs reared for fattening are slaughtered at the age of 5 months.

### Sample collection

The minimum sample size was calculated to be 246 pigs using Win Episcope 2.0. Criteria for sample size calculation included a confidence level of 95%, an accepted error of 5%, a population size of 30,000 (around 25,000 pigs were slaughtered in 2018; [22], and a minimum expected prevalence of 20% (The prevalence of animal brucellosis in Egypt is between 0.2% and 20%; [15]. A total of 1,116 slaughtered pigs were randomly selected from 19 February to 10 December 2020. The pigs from various herds are collected together in abattoir pens before slaughtering without any identification. Most of these animals are bought by pig merchants who collected them from different small-scale farmers before shipping them to the abattoir. For this reason, it was impossible to trace back sampled slaughtered pigs to their origin herds. Cervical lymph nodes (LN), inguinal LN, and supra-mammary LN were collected for each pig. In addition, the gender and any gross pathological lesions of chosen pigs' carcasses were recorded. All samples were labeled and transferred on ice to the Reference Laboratory of Brucellosis Research at the Animal Health Research Institute (AHRI), Giza, for *Brucella* isolation and identification.

### Isolation of brucellae and identification of species and biovars

Swaps of macerated lymph nodes were cultured on trypticase soy agar (Oxoid LTD, Basingstoke, UK) supplemented with *Brucella* selective supplement (Oxoid LTD, Basingstoke, UK). The culture plates were incubated with and without a 5–10% CO_2_ atmosphere at 37 °C and examined for *Brucella* growth for two weeks.

Phenotypic characterization of the *Brucella* isolates was conducted as previously described [30] in terms of colony morphology, urease, catalase, oxidase, nitrate reduction, lactose fermentation, and reaction to acriflavine (1/1,000 solution) to detect dissociation and ensure smoothness of colonies (genus identification). All suspected *Brucella* isolates were sent to the Institute of Bacterial Infections and Zoonoses (IBIZ, Jena, Germany) for molecular confirmation of *B. suis* isolates at the species and the biovar levels. In addition, Matrix-Assisted Laser Desorption/Ionization (MALDI-TOF–MS) was performed as previously described [36] for rapid *Brucella* genus identification. A safe genus identification' was defined as a MALDI log score between 2.000 and 2.290. Furthermore, the requirement for CO_2_ at initial culture, H_2_S production, growth in the presence of dyes (thionine at 1/25,000 concentration; basic fuchsin at 1/50,000; safranin O dye 100 µg/ml), agglutination with A, M, and R monospecific anti-sera, and lysis by Tbilisi (Tb) phage using both routine test dilution (RTD) and 10^4^ × RTD and Izatnagar (Iz) phage were done for conventional identification of *Brucella* species and biovar.

### Molecular confirmation of B. suis and its biovars

The conventionally identified *Brucella* isolates were molecularly confirmed at the genus level using IR-1 and IR-2 primers as previously described [37]. The *Brucella* species were identified using AMOS-PCR [37] and Bruce-Ladder multiplex PCR [38]. The *B. suis* biovar was confirmed using Suis-ladder multiplex PCR [39].

### Defining high-risk practices of swine abattoir workers

High-risk practices of 16 abattoir workers (4 veterinarians, 10 butchering and evisceration workers, and 2 scalding workers) were investigated using a pre-piloted structured questionnaire. The abattoir workers' practices included questions about using personal protective equipment (PPE), working with open wounds on their hands, eating or smoking while working, hand washing habits, and routine disinfection of knives.

### Statistical analysis

The Univariate regression analysis was carried out using SPSS v19 (IBM, Armonk, NY). A significant association was considered at *P* ≤ 0.05.

## Supplementary Information

**Additional file 1:** **Supplementary Table 1.** Detailed identification of Brucella suis biovar 2 recovered from cervical lymph nodes of slaughtered pigs at El-Basatin abattoir, Egypt during 2020 using MALDI-TOFMS, classical bacteriological typing, AMOS-PCR, and B. suis ladder PCR. **Supplementary Table 2.** Pre-piloted structured questionnaire template used to collect data regarding abattoir workers' high-risk practices.

## Data Availability

All data generated or analyzed during this study are included in this published article and its supplementary information files.

## Abbreviations

AHRI: Animal Health Research Institute
Biovar: Bv
Brucella: B
CI 95%: 95% Confidence intervals
FAOSTAT: The Food and Agriculture Organization Corporate Statistical Database
GOVS: General Organization of Veterinary Services
IBIZ: Institute of Bacterial Infections and Zoonoses
Iz: Izatnagar phage
LN: Lymph node
MALDI-TOF–MS: Matrix-Assisted Laser Desorption/Ionization Time-of-Flight Mass Spectrometry
OR: Odds ratio
PCR: Polymerase chain reaction
PPE: Personal protective equipment
Prevalence: *P*
RTD: Routine test dilution
Tb: Tbilisi phage
WGS: Whole Genome Sequencing
