# Supplementary material for: Isolation and molecular confirmation of Brucella suis biovar 2 from slaughtered pigs: an unanticipated biovar from domestic pigs in Egypt
Source: BMC Vet Res. 2022 Jun 13;18:224. doi: 10.1186/s12917-022-03332-2 (PMC9195200; doi:10.1186/s12917-022-03332-2)
Supplement: Supplementary file 1 — Additional file 1: Supplementary Table 1. Detailed identification of Brucella suis biovar 2 recovered from cervical lymph nodes of slaughtered pigs at El-Basatin abattoir, Egypt during 2020 using MALDI-TOFMS, classical bacteriological typing, AMOS-PCR, and B. suis ladder PCR. Supplementary Table 2. Pre-piloted structured questionnaire template used to collect data regarding abattoir workers' high-risk practices. [file 12917_2022_3332_MOESM1_ESM.pdf]

Isolation and molecular confirmation of *Brucella suis* biovar 2 from slaughtered pigs: an unanticipated biovar from domestic pigs in Egypt

Walid Elmonir, Nour H. Abdel-Hamid, Mahmoud E. R. Hamdy, Eman I. M. Beleta, Mohamed El-Diasty, Falk Melzer, Gamal Wareth & Heinrich Neubauer

Additional file 1:

Supplementary Table 1. Detailed identification of *Brucella suis* biovar 2 recovered from cervical lymph nodes of slaughtered pigs at El-Basatin abattoir, Egypt during 2020 using MALDI-TOFMS, classical bacteriological typing, AMOS-PCR, and *B. suis* ladder PCR.

Input	ID	Sample	Host	Original number	Country	Genus Brucella PCR	AMOS-PCR	MALDITOF	Classical Bacteriology	*B. suis* Ladder PCR
**08.04.**	**21RB22953**	Isolate	Pig	11; 2/2020; group 1	Egypt	*Brucella*	Not identified	*Brucella* spp	*Brucella suis*	*Brucella suis 2*
**08.04.**	**21RB22954**	Isolate	Pig	16; 16/7/2020; group 2	Egypt	*Brucella*	Not identified	*Brucella* spp	*Brucella suis*	*Brucella suis 2*
**08.04.**	**21RB22955**	Isolate	Pig	18; 26/7/2020; group 3	Egypt	*Brucella*	Not identified	*Brucella* spp	*Brucella suis*	*Brucella suis 2*
**08.04.**	**21RB22956**	Isolate	Pig	32; 26/7/2020; group 3	Egypt	*Brucella*	Not identified	*Brucella* spp	*Brucella suis*	*Brucella suis 2*
**08.04.**	**21RB22957**	Isolate	Pig	20; 26/7/2020; group 3	Egypt	*Brucella*	Not identified	*Brucella* spp	*Brucella suis*	*Brucella suis 2*
**08.04.**	**21RB22958**	Isolate	Pig	3; 18/10/2020; group 5	Egypt	*Brucella*	Not identified	*Brucella* spp	*Brucella suis*	*Brucella suis 2*
**08.04.**	**21RB22959**	Isolate	Pig	4; 18/10/2020; group 5	Egypt	*Brucella*	Not identified	*Brucella* spp	*Brucella suis*	*Brucella suis 2*
**08.04.**	**21RB22960**	Isolate	Pig	11; 18/10/2020; group 5	Egypt	*Brucella*	Not identified	*Brucella* spp	*Brucella suis*	*Brucella suis 2*
**08.04.**	**21RB22961**	Isolate	Pig	17; 18/10/2020; group 5	Egypt	*Brucella*	Not identified	*Brucella* spp	*Brucella suis*	*Brucella suis 2*
**08.04.**	**21RB22962**	Isolate	Pig	23; 18/10/2020; group 5	Egypt	*Brucella*	Not identified	*Brucella* spp	*Brucella suis*	*Brucella suis 2*
**08.04.**	**21RB22963**	Isolate	Pig	6; 18/10/2020; group 5	Egypt	*Brucella*	Not identified	*Brucella* spp	*Brucella suis*	*Brucella suis 2*
**08.04.**	**21RB22964**	Isolate	Pig	16; 22/10/2020; group 6	Egypt	*Brucella*	Not identified	*Brucella* spp	*Brucella suis*	*Brucella suis 2*
**08.04.**	**21RB22965**	Isolate	Pig	5; 1/12/2020; group 7	Egypt	NB	Not identified	*Ochrobacter intermedium*	Ochrobacter	ND
**08.04.**	**21RB22966**	Isolate	Pig	5; 10/12/2020; group 8	Egypt	NB	Not identified	Not identified	Not Brucella	ND
**08.04.**	**21RB22967**	Isolate	Pig	21; 26/7/2020; group 3	Egypt	*Brucella*	Not identified	*Brucella* spp	*Brucella suis*	*Brucella suis 2*
**08.04.**	**21RB22968**	Isolate	Pig	26; 22/10/2020; group 6	Egypt	*Brucella*	Not identified	*Brucella* spp	*Brucella suis*	*Brucella suis 2*
**08.04.**	**21RB22969**	Isolate	Pig	18; 5/9/2020; group 4	Egypt	NB	Not identified	Not identified	Not *Brucella*	ND
**08.04.**	**21RB22970**	Isolate	Pig	From 14; 10/12/2020; group 8	Egypt	NB	Not identified	Not identified	Not *Brucella*	ND
**08.04.**	**21RB22971**	Isolate	Pig	From 16; 22/10/2020; group 6	Egypt	NB	Not identified	Not identified	Not *Brucella*	ND

ND; Not done, NB; *Not Brucella*

Additional file 2

Supplementary Table 2. Pre-piloted structured questionnaire template used to collect data regarding abattoir workers' high-risk practices.

**(A) Identification:**
**1- Name (optional):**
**2- Age:**
**3- Gender:**
*Male *Female
**4- Education:**
*No *Primary *Secondary *Higher
**5- Residence:**
*Urban *Rural
**(B) Occupational exposure information:**
**6– What is your occupation?**
*Vet *Butcher *Cleaner *Other: ……………..…
**7– How long have you been working?**
………………………..………..…(Years)
**8– Do you use protective closing?**
*Yes *No
**9– If yes, What kind of protective clothing?**
*Mask *Gloves *Googles *Face shield *Boots *Apron
**10– Do you work with open/cut hand wound?**
*Yes *No
**11– Do you eat while working?**
*Yes *No
**12– Do you smoke while working?**
*Yes *No
**13– Do you wash your hand before eating or smoking?**
*Yes *No
**14– Do you wash your hand after work?**
*Yes *No
